# Study on Mass and Performance Deterioration of Concrete Under Multiple Corrosive Environments

**DOI:** 10.3390/ma18091931

**Published:** 2025-04-24

**Authors:** Haicheng Yang, Weifeng Liu, Hongfa Yu, Wei Wang, Haiyan Ma

**Affiliations:** 1 CCCC Fourth Harbor Engineering Institute Co., Ltd., Guangzhou 510230, China; yhaicheng@gzpcc.com (H.Y.); 2022015508@ccccltd.cn (W.W.); 2 Department of Civil and Airport Engineering, Civil Aviation College, Nanjing University of Aeronautics and Astronautics, Nanjing 210016, China; mahaiyan@nuaa.edu.cn

**Keywords:** solution corrosion, dry–wet cycling, mass loss, relative dynamic modulus, slag

## Abstract

This study investigates the corrosion behavior, mass changes, and relative dynamic elastic modulus variations of PC (Portland cement concrete) and G60 (with 60% slag) under two treatment methods: immersion in 3.5% NaCl solution and composite (3.5% NaCl + 5.7% Na_2_SO_4_) solution, as well as dry–wet cycles. Results indicate that under water and NaCl conditions, no significant mass loss or modulus reduction occurred, with only minor surface efflorescence. However, in the composite solution, severe spalling was observed, with mass loss reaching approximately 1.4% and the relative dynamic modulus decreasing to around 90% after 540 days. Dry–wet cycling accelerated the corrosion process compared to immersion, leading to greater mass loss and modulus reduction. The incorporation of slag improved resistance to ionic penetration in both chemical corrosion and salt crystallization, though this effect diminished with prolonged exposure or increased cycling.

## 1. Introduction

Concrete, as the most widely used construction material, is extensively employed in buildings, bridges, and infrastructure projects [1,2,3]. Its diverse service environments necessitate careful consideration of durability during the design phase. Structural failures caused by durability issues have led to numerous cases where projects did not achieve their intended lifespan, requiring extensive repairs. Examples include the damage to the Graus and Tavascán dams in Spain [4,5], housing defects in Donegal County, Ireland [6,7], foundation failures in Connecticut, USA [8], and the collapse of commercial buildings in Trois-Rivières in Canada due to rapid degradation of concrete foundations [9]. These durability issues have resulted in substantial economic and societal losses.

Major durability issues in large-scale engineering projects have garnered significant attention from researchers worldwide. There is an urgent need to optimize mix designs while considering practical conditions to ensure durability and achieve the intended service life of these structures.

Sulfate attack is a common phenomenon affecting concrete in natural environments, involving complex physicochemical processes. These include chemical alterations of concrete components (e.g., formation of gypsum and ettringite) and physical erosion caused by volumetric expansion during the hydration of sulfates [10]. Chemical corrosion of cementitious materials by sulfates is the predominant mechanism. When the cations in the corrosive medium do not react with the hydration products of concrete, and sulfate ions (SO_4_^2−^) are the primary corrosive agents, the mechanism involves the diffusion of SO_4_^2−^ into the concrete matrix. The ions react with hydration products to form expansive compounds such as gypsum (CaSO_4_·2H_2_O) and ettringite (Aft, 3CaO·Al_2_O_3_·3CaSO_4_·32H_2_O). The relevant reactions are detailed in Equations (1)–(3) [11]. When the resulting expansion stress exceeds the tensile strength of concrete, microcracks develop, leading to strength loss, surface spalling, and eventual structural failure. Typical damage manifests as internal cracking due to expansion or progressive surface erosion.

(1) $$Ca{\left(OH\right)}_{2}+Na_{2}SO_{4}+2H_{2}O\to CaSO_{4}\cdot 2H_{2}O+2NaOH$$

(2) $$\begin{array}{l} 2(CaSO_{4}\cdot 2H_{2}O)+3CaO\cdot Al_{2}O_{3}\cdot CaSO_{4}\cdot 12H_{2}O+16H_{2}O\\ \to 3CaO\cdot Al_{2}O_{3}\cdot 3CaSO_{4}\cdot 32H_{2}O \end{array}$$

(3) $$3(CaSO_{4}\cdot 2H_{2}O)+3CaO\cdot Al_{2}O_{3}+26H_{2}O\to 3CaO\cdot Al_{2}O_{3}\cdot 3CaSO_{4}\cdot 32H_{2}O$$

Manu Santhanam et al. [12] investigated the expansion and microstructure of different Portland cement mortars in Na_2_SO_4_ solution, identifying nucleation and delayed ettringite formation as the primary causes of volumetric expansion due to sulfate attack. Their findings indicate that concrete with smaller capillary pore sizes exhibits better resistance to sulfate corrosion. Additionally, an increase in the temperature and concentration of the solution significantly reduces concrete’s resistance to sulfate attack. To address sulfate-induced degradation, Gollop R S et al. [13] innovatively designed sulfate-resisting Portland cement (SRPC). Microstructural analysis revealed that ettringite had already formed prior to exposure to the corrosive solution, resulting in a marked reduction in the formation of ettringite induced by external sulfate ions and a consequent decrease in expansion.

In practical service environments, concrete structures are often exposed to multiple harmful ions, including SO_4_^2−^, Cl^−^, and CO_3_^2−^. Current research primarily focuses on the diffusion of SO_4_^2−^ through pore solution under hydrostatic pressure, where it reacts with hydration products to form expansive compounds. The diffusion of Cl^−^ into concrete can also lead to the failure of the passive film on reinforcement, causing expansion and degradation of the concrete cover [14,15,16,17,18]. However, these harmful ions typically do not exist independently; they interact, and can exhibit both synergistic and antagonistic effects. To study the chemical corrosion of concrete in a chloride–sulfate mixed ion environment, understanding the interactions between sulfate and chloride ions is essential. Progress has been made in this area of research.

Nie et al. [19] developed a dynamic statistical damage stress–strain model for corroded concrete based on the Weibull distribution theory, investigating the corrosion of concrete under the combined effect of chloride and sulfate solutions. The study revealed the mechanism by which sulfates affect the stability of chloride ions in cement paste. T. Chiker et al. [20] analyzed the impact of the water-to-cement ratio and silica fume on the durability of concrete exposed to sulfate and chloride mixed solutions, finding that chloride ions had a more significant effect on the sulfate content of concrete with high and medium water-to-cement ratios. Du et al. [21] used XRD and SEM techniques to uncover the key inhibitory mechanisms by which external chlorides mitigate sulfate-induced corrosion of concrete. Yu et al. [22] applied a combined physical-chemical-mechanical approach to investigate the composite corrosion model of sulfate–chloride interaction in concrete, offering new diversified methods for future engineering practice research.

In practical engineering, concrete durability is also influenced by environmental and climatic factors. In regions with fluctuating water levels, such as splash zones and tidal areas, in addition to ion corrosion, the effects of dry–wet cycles must also be considered [23,24,25]. In corrosive environments, dry–wet cycles primarily cause severe salt crystallization damage to concrete. The deterioration of concrete durability under dry–wet cycling can be categorized into two types: chemical corrosion and physical erosion [26]. Chemical corrosion results from reactions between salts in the corrosive medium and cement hydration products, leading to concrete damage. Physical erosion occurs when concrete structures in fluctuating water level environments absorb soluble salt solutions through capillary action during wet conditions. As the structure dries, water evaporates, and the salt solution in the pores becomes saturated, leading to the crystallization and concentration of soluble salts. The resulting crystallization pressure causes concrete degradation [27]. However, the crystallized salts do not chemically react with the cement hydration products, representing a physical rather than chemical process.

According to Seherer et al. [28], the crystallization pressure exerted on the pore walls of concrete by salt crystallization primarily results from several factors: phase volume expansion caused by the transformation of different crystal forms of the same salt, hydration pressure from water absorption by anhydrous salts, expansive forces from the growth of hydration products, and hydrostatic pressure. Nicholas T et al. [29] experimentally confirmed that the volumetric expansion caused by crystal transformation in concrete pores generates crystallization pressure on the pore walls, which is the main cause of concrete degradation in corrosive environments. Additionally, an increase in temperature amplifies this pressure.

Yang et al. [30] used Na_2_SO_4_ as a corrosive medium to conduct both solution immersion and dry–wet cycle tests, finding that salt crystallization corrosion was more severe under dry–wet cycle conditions. As the solution concentration and number of dry–wet cycles increased, concrete exhibited more pronounced expansion. Wang et al. [31] studied the relationship between erosion depth and time under dry–wet sulfate cycles and proposed a method to predict long-term erosion based on short-term results. The findings indicated that for the same erosion depth, a shorter erosion time corresponded to faster erosion, and concrete with a circular interface had higher permeability than that with a rectangular interface. Su et al. [32] conducted immersion and dry–wet cycle tests in sulfate and chloride salt solutions, using mass loss and dynamic elastic modulus changes as references. The results showed that corrosion damage to concrete was exacerbated under dry–wet cycling conditions. Guo et al. [24] performed four dry–wet cycle tests (3 d, 7 d, 14 d, and 21 d) in sulfate environments, measuring bending strength, relative dynamic modulus, and mass. The results revealed that as the dry–wet cycle duration increased, concrete deterioration initially increased and then decreased. However, excessively prolonging the dry–wet period did not significantly exacerbate concrete performance degradation.

In order to resist the damage caused by sulfate attack on concrete, a variety of means have been adopted, including the addition of mineral admixtures [33,34,35,36,37,38,39], the use of lightweight aggregates and viscosity modifiers [40], the addition of glass powder [41,42] and the use of plasticizers [33] or carbonates [43]. In view of the easy availability and superior performance of mineral admixtures, they are most widely used. Among them, with the increase of slag content, the ability of concrete to resist sulfate attack will also be enhanced, but beyond a certain range, there will be a trend of performance degradation.

Rudra P. S. et al. [44] studied the effect of 50% slag content on the sulfate resistance of concrete, and found that it greatly improved the sulfate resistance of concrete without significant change in strength. With the further increase of the content, the research of J. Stroh et al. [45] shows that when the content of slag is as high as 80%, although the ability of concrete to resist sulfate attack is significantly improved, it has a great influence on the performance of concrete itself. Therefore, this paper only chooses 60% of the slag content for research, hoping to give concrete a stronger ability to resist sulfate attack on the basis of retaining the mechanical properties of concrete itself to the greatest extent.

In view of the fact that the time required for concrete durability test is generally long, in order to accelerate the test process, a series of accelerated test methods have appeared, such as the internal adding method. This kind of accelerated test method has certain reference value and significance, but there is still a big difference with the actual environment [46,47]. In addition, there is currently no non-destructive experimental indicators such as mass loss and relative dynamic elastic modulus are used as criteria for judging the change of performance to study the durability of concrete for a long time. Since the specimens are not damaged, this test method is convenient for subsequent data and laws of longer corrosion time.

Therefore, this study designed two concrete mixes with different proportions, using 3.5% NaCl solution and a composite solution (3.5% NaCl + 5.7% Na_2_SO_4_) as corrosive media. Long-term natural immersion and dry–wet cycle tests were conducted to explore the chemical corrosion and salt crystallization corrosion characteristics of concrete in chloride solutions and chloride–sulfate mixed solutions. The study also examined changes in the mass and relative dynamic modulus of elasticity during the damage process. Additionally, the effect of slag as a mineral admixture on enhancing concrete’s resistance to ionic corrosion was evaluated, providing insights for mix design under multiple corrosive environmental conditions.

## 2. Experiment

### 2.1. Sample Preparation

#### 2.1.1. Raw Materials

The cement used was P. II 52.5R Portland cement produced by Jiaxin Jingyang Cement Co., Ltd. (Zhenjiang, China). Its chemical composition is detailed in Table 1, and key physical and mechanical properties are listed in Table 2.

The slag used was S95-grade ground granulated blast furnace slag produced by Jiangsu Jiangnan Grinding Co., Ltd. (Nanjing, China). Its chemical composition is detailed in Table 3.

**Figure 1:**
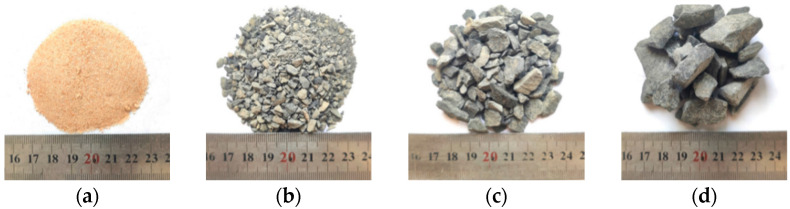
Coarse and fine aggregates. (**a**) Fine sand; (**b**) Manufactured sand; (**c**) 3~8 mm crushed stones; (**d**) 8~16 mm crushed stones.

Crushed stones, manufactured sand, and fine sand with different particle sizes were used as coarse and fine aggregates for concrete preparation, as shown in Figure 1. Their basic physical properties are detailed in Table 4.

#### 2.1.2. Mix Proportions

Two different concrete mix proportions were designed in this study, optimizing aggregate grading and cement paste design to improve the workability, mechanical properties, and resistance to chloride and sulfate ion corrosion of the concrete. The influence of different types of cementitious materials on concrete performance was also analyzed. The specific mix proportions are detailed in Table 5. In the mix, the mass fraction of 8–16 mm crushed stone was 66%, the replacement rate of fine sand with manufactured sand was 50%, the sand-to-cement ratio was 37%, and the water-to-cement ratio was 0.38. The final specimens were prepared with dimensions of 150 mm × 150 mm × 150 mm.

### 2.2. Experiment Methods

#### 2.2.1. Corrosion Test Scheme

**Figure 2:**
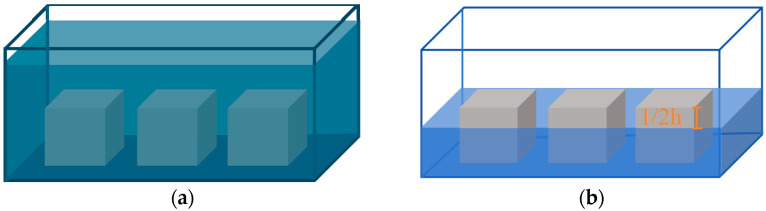
Schematic diagram of specimen solution corrosion and dry–wet cycling. (**a**) Solution corrosion; (**b**) Dry–wet cycling.

The corrosive media used in this experiment were 3.5% NaCl solution and a composite solution of 3.5% NaCl + 5.7% Na_2_SO_4_, both prepared using chemical reagents, with distilled water as the control group. The chemical corrosion environment was simulated by long-term natural immersion, with the solution completely submerging the specimens. The immersion times selected were 0 d, 30 d, 60 d, 120 d, 180 d, 270 d, 330 d, 360 d, 390 d, 460 d, 500 d, and 540 d. In addition to natural immersion, concrete often appears coupled in a dry–wet cycle in a sulfate environment. By referring to the test method of dry–wet cycle of sulfate erosion in the ‘Test method for long-term performance and durability of ordinary concrete’ (GB/T 50082-2009) [48], a natural dry–wet cycle method was employed with a 7-day cycle for salt crystallization corrosion. Half of the specimen was submerged in the corrosive solution, and the other half exposed to the air; after 3.5 days, the specimen was rotated. This process was repeated for cycles of 0, 5, 8, 15, 25, 38, 47, 51, 56, 66, 72, and 77 cycles. A schematic diagram of the specimen treatment is shown in Figure 2.

#### 2.2.2. Experimental Testing Methods

(1) Mass loss

Under the influence of corrosive ions, concrete surfaces are prone to spalling, leading to changes in overall weight. Therefore, mass loss can serve as an indicator of surface spalling. When the immersion time or dry–wet cycle count reaches the set criteria, the specimens should be removed from the solution, rinsed with distilled water, and the surface wiped. Then, the mass change of the concrete specimen during corrosion is measured using an electronic balance with an accuracy of 0.1 g. The mass loss rate is calculated according to Equation (4):

(4) $$W_{l}=\frac{G_{t}-G_{0}}{G_{0}}\times 100\%$$

In the equation, *W_l_* is the mass change rate of the specimen after corrosion (%), with a positive value indicating an increase in concrete mass and a negative value indicating mass loss.

*G*_0_ is the mass of the specimen before corrosion (g), and *G_t_* is the mass of the specimen after corrosion (g).

(2) Relative dynamic elastic modulus

**Figure 3:**
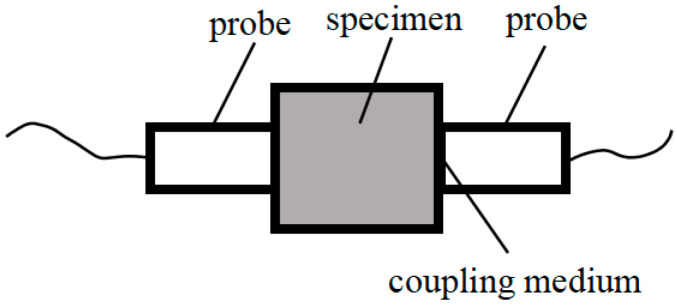
Schematic diagram of ultrasonic testing.

The methods commonly used by researchers to characterize concrete damage in corrosive environments primarily focus on qualitative and quantitative studies using surface morphology or strength data. However, surface morphology is too subjective, and strength data is inherently discrete, with strength testing being a destructive method not suitable for long-term durability tests, especially when specimens are valuable. Therefore, there is an urgent need to identify new detection methods or indicators to assess the damage to concrete. Ultrasonic testing is a novel non-destructive technique that can determine the extent of damage in concrete by measuring the elastic velocity and wave amplitude, without causing damage to the specimens. This makes it highly suitable for continuous monitoring of concrete damage throughout the corrosion process. In this experiment, ultrasonic testing was used to measure the concrete’s acoustic pulse time, from which the relative dynamic elastic modulus was calculated [49] (as shown in Equation (5)), to assess the internal damage of the concrete. The equipment used for testing was the NM-4B non-metallic ultrasonic testing and analysis instrument, manufactured by Beijing Kangkerui Company (Beijing, China), with a measurement range of 150 mm. A schematic diagram of the testing setup is shown in Figure 3.

(5) $$E_{rd}=\frac{v_{n}^{2}}{v_{0}^{2}}=\frac{t_{0}^{2}}{t_{n}^{2}}\times 100\%$$

where, *v*_0_ and *v_n_* are the sound velocities of the specimen before and after corrosion; *t*_0_ and *t_n_* are the ultrasonic pulse times of the specimen before and after corrosion.

## 3. Results and Discussion

### 3.1. Chemical Corrosion

#### 3.1.1. Mass Change Under Chemical Corrosion Conditions

**Figure 4:**
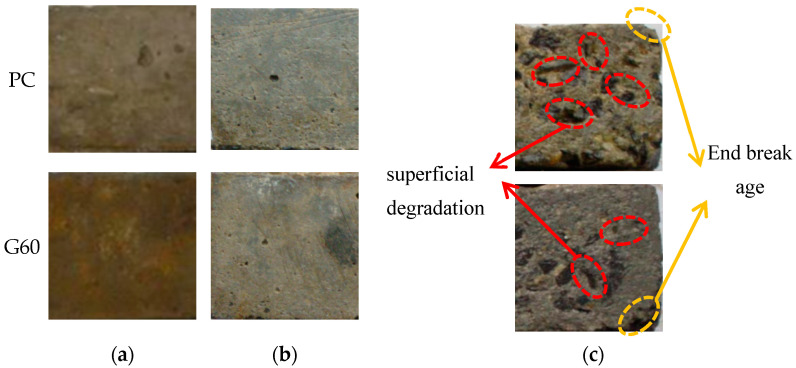
Corrosion morphology of concrete after 540 days of immersion in different solutions. (**a**) Distilled water; (**b**) 3.5% NaCl solution; (**c**) 3.5% NaCl + 5.7% Na_2_SO_4_ composite solution.

Figure 4 illustrates the failure morphology of PC and G60 concretes after immersion in different solutions for up to 540 days. Specimens immersed in distilled water and NaCl solution show minimal visible surface damage. In contrast, those exposed to the composite solution exhibit significant surface degradation, including roughness, surface scaling, mortar disintegration, aggregate exposure, and in some cases, end-face damage.

The mass variation data for PC and G60 concretes immersed in distilled water, 3.5% NaCl solution, and the composite solution are shown in Table 6. Negative values indicate mass loss. The data clearly reveal that mass loss occurs earlier for concrete specimens in the composite solution compared to those in distilled water and 3.5% NaCl solution, highlighting the more severe damage caused by the combined action of sulfates and chlorides.

**Figure 5:**
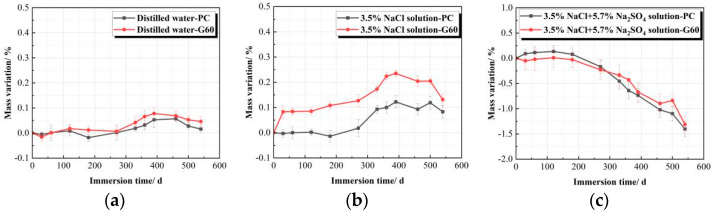
Mass variation trends of concretes in different solutions. (**a**) Distilled water; (**b**) 3.5% NaCl solution; (**c**) Composite solution.

When the data are summarized over time and plotted in Figure 5, it is evident that both types of concrete initially exhibit an increase in mass due to water absorption in the early immersion stages, reaching a peak at approximately 400 days. Subsequently, fluctuations are observed, but the overall trend declines. At 540 days, no mass loss is detected in distilled water and 3.5% NaCl solutions. The mass gain rates are 0.016% and 0.046% in distilled water and 0.083% and 0.131% in 3.5% NaCl solution for PC and G60 concretes. The higher mass gain in the chloride solution is primarily attributed to salt crystallization on the concrete surface.

In contrast, immersion in the composite solution for 540 days resulted in significant mass loss for both PC and G60, with G60 exhibiting a lower mass loss rate of 1.266% and PC showing a higher rate of 1.403%. During the initial stages, PC experienced greater mass gain compared to G60. However, as the immersion period progressed, both concretes began losing mass at around 200 days. PC exhibited a faster cumulative mass loss, surpassing G60 after 300 days. This difference is attributed to the denser structure of G60 due to slag addition, which led to lower initial water absorption and reduced spalling from erosion in later stages. In contrast, PC showed higher mass gain initially but suffered more severe erosion-induced spalling over time.

**Figure 6:**
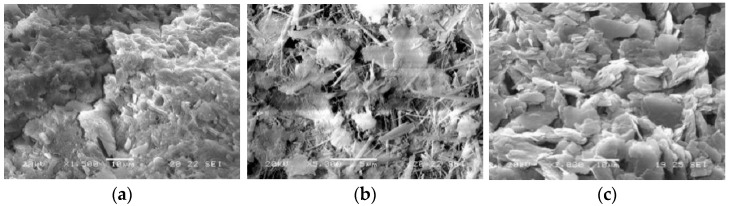
SEM micrographs of expansive products in composite solution. (**a**) CaSO_4_·2H_2_O; (**b**) AFt; (**c**) AFm.

**Figure 7:**
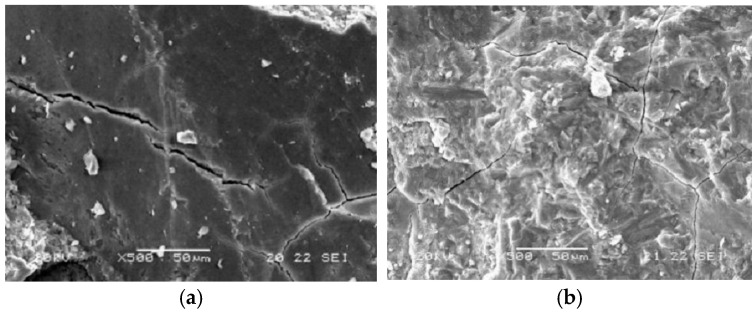
Microcracks in PC and G60 immersed in the composite solution. (**a**) Microcracks in PC immersed in the composite solution; (**b**) Microcracks in G60 immersed in the composite solution.

Comparative analysis revealed that NaCl solution caused negligible mass loss in concrete, whereas the composite solution of sulfate and chloride ions led to significant mass loss for both concrete types. SEM observations (Figure 6) indicate that the presence of sulfates resulted in the formation of expansive corrosion products, including needle-shaped gypsum (CaSO_4_·2H_2_O), ettringite (AFt), and monosulfate (AFm). These products contributed to the development of microcracks within the concrete and exacerbated surface spalling, ultimately manifesting as mass loss, as depicted in Figure 7a.

The incorporation of slag can combine with calcium hydroxide in cement, forming hydration products. This process not only densifies the internal structure of the concrete, thereby resisting the penetration of sulfate ions (as shown in Figure 7b), but also reduces the likelihood of sulfate reacting with calcium hydroxide to form gypsum, thereby preventing the subsequent formation of expansive products like ettringite. Consequently, the data show that the G60 with slag has a lower mass loss rate.

#### 3.1.2. Relative Dynamic Elastic Modulus (E*_rd_*) Variation Under Chemical Corrosion Conditions

**Figure 8:**
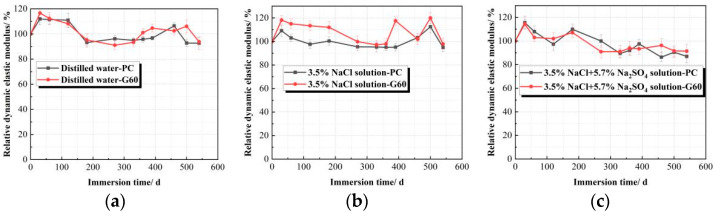
E*_rd_* variation trends of concretes in different solutions. (**a**) Distilled water; (**b**) 3.5% NaCl solution; (**c**) Composite solution.

Table 7 and Figure 8 show the E*_rd_* experimental data and trends for both concrete types in three different solutions. The results reveal that the E*_rd_* of concrete immersed in water and 3.5% NaCl solution fluctuates throughout the experiment without significant decline, maintaining a minimum value above 90%. This suggests that NaCl solution does not impair the concrete’s performance. Conversely, in the composite solution containing chloride and sulfate salts, the E*_rd_* initially increases slightly before decreasing. During early corrosion, sulfate ions diffuse into the near-surface region of the concrete, forming ettringite and densifying the structure [50], which increases E*_rd_*. However, as corrosion progresses, ettringite crystals grow, generating expansive stresses that exceed the concrete’s tensile strength, leading to microcracks. Subsequently, rapid penetration of sulfate ions forms more ettringite and gypsum, causing significant internal damage and a reduction in E*_rd_* After 540 days of immersion, the E*_rd_* of PC and G60 concrete decrease to 86.992% and 91.380%.

Compared to PC, the G60 blended with slag exhibits stronger resistance to sulfate ion erosion. This is primarily due to the greater fineness of slag compared to cement, which can fill the gaps between cement particles, achieving a dense filling structure and a self-compacting packing system at the microscopic level, thereby reducing the permeability of sulfate ions. Additionally, due to the volcanic ash activity of slag, it can react with Ca(OH)_2_ in the concrete, generating additional hydration products, which not only fill the concrete structure but also reduce the formation of substances such as gypsum and ettringite.

### 3.2. Salt Crystallization Corrosion Under Dry–Wet Cycling

#### 3.2.1. Mass Changes Under Salt Crystallization Conditions

**Figure 9:**
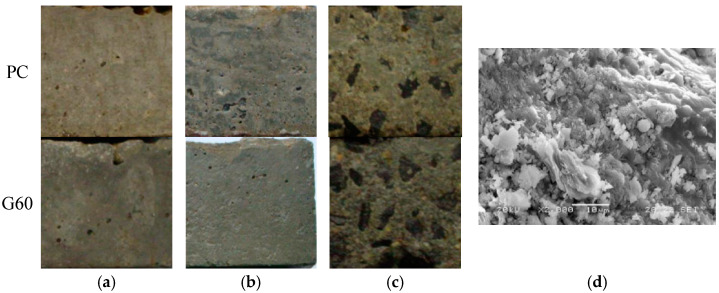
Corrosion morphology of concrete under 77 dry–wet cycles in different solutions. (**a**) Distilled water; (**b**) 3.5% NaCl solution; (**c**) 3.5% NaCl + 5.7% Na_2_SO_4_ composite solution; (**d**) Surface-exposed sodium chloride on the specimen.

Figure 9 shows the damage morphology of two types of concrete under dry–wet cycling conditions in three different solutions. From the figure, it can be observed that at the end of the experiment, the surface of the specimens in 3.5% NaCl solution appeared white after dry–wet cycling. This whiteness is due to the formation of white NaCl crystals at the end exposed to the air. However, the corrosion degree of the concrete itself is relatively mild, and compared to G60, while the PC surface shows slight powdering and appears somewhat rough. This indicates that although chloride salt crystallization corrosion does not cause expansion or cracking damage inside the concrete, it results in slight spalling damage on the concrete surface. Since salt crystallization corrosion is closely related to capillary phenomena and ion diffusion and penetration under dry–wet cycling, the chloride salt concentration accumulated on the surface layer of the concrete increases with the number of cycles, leading to greater crystallization pressure. Only when the dry–wet cycles reach a certain number will the salt crystals continue to accumulate and grow in the dense concrete pores, generating expansion stress. At this point, the rate of salt crystallization damage will accelerate, and the deterioration effect on the concrete will gradually become apparent. The main damage characteristic is the expansion of the concrete and surface spalling damage, which typically does not cause internal damage.

Under the composite solution salt crystallization corrosion conditions, the surface spalling of both concrete types is severe, with large areas of exposed aggregates and significant damage to the ends and corners. In contrast, the surface spalling of G60 is not as severe, but most of the surface is powdered, with mortar spalling, exposed aggregates, and damage beginning to appear at the ends and corners.

**Figure 10:**
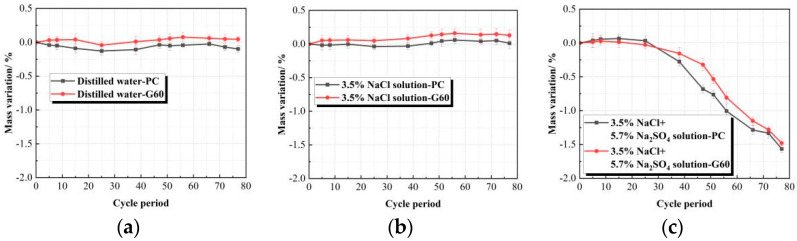
Mass variation trends of concretes under dry–wet cycling. (**a**) Distilled Water; (**b**) 3.5%NaCl Solution; (**c**) Composite Solution.

Table 8 and Figure 10 show the mass change rate and time curve of the two types of concrete under salt crystallization corrosion conditions in three different solutions. From the combined analysis, it can be seen that after 77 consecutive dry–wet cycles (corresponding to 540 days of soaking) in distilled water and 3.5% NaCl solution, the mass change trends of the specimens are generally consistent, with no mass loss observed. Furthermore, the G60 specimens exhibit an increase in mass, with overall changes similar to those under solution corrosion conditions.

From Table 8, it can be seen that after 77 dry–wet cycles under different solution conditions, the mass loss of concrete in the composite solution reaches approximately 0.6%. With the increase in dry–wet cycles, the mass change of G60 first increases and then decreases, reaching a peak after 15 dry–wet cycles, followed by a gradual decrease. As the number of dry–wet cycles increases, the mass change differences between the two types of concrete gradually diminish, becoming nearly the same after 77 cycles. This indicates that under fewer dry–wet cycles, adding slag can effectively reduce the erosion of concrete by sulfate, but this effect gradually diminishes with the increase in cycles, eventually showing the same properties as ordinary concrete without slag.

By comparing with Figure 5, it can be observed that the mass loss of concrete under 77 dry–wet cycles is greater than the mass loss under the corresponding 540 days of solution corrosion conditions. This suggests that salt crystallization accelerates the corrosion and deterioration of concrete in sulfate and chloride composite solutions, with a significant impact on surface spalling and damage.

#### 3.2.2. Relative Dynamic Elastic Modulus (E*_rd_*) Variation Under Salt Crystallization Conditions

**Figure 11:**
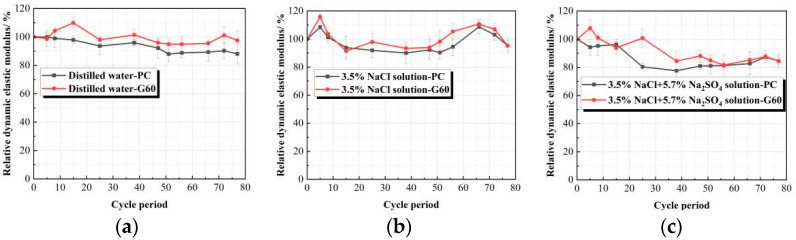
E*_rd_* variation trends of concretes under dry–wet cycling. (**a**) Distilled Water; (**b**) 3.5%NaCl Solution; (**c**) Composite Solution.

The variation pattern and curve of the E*_rd_* of concrete under dry–wet cycling in different solutions are shown in Table 9 and Figure 11. By examining the data in the table and the curve changes in the figure, it can be observed that the E*_rd_* of the specimens under dry–wet cycling in all three solutions exhibits a trend of first increasing and then decreasing. Unlike the specimens in clear water and composite solutions, where the E*_rd_* shows a brief increase after 56 cycles, the specimens in the sodium chloride solution exhibit a sharp decline after 66 cycles. However, since chloride salts do not chemically corrode concrete, under the combined action of sodium chloride solution and dry–wet cycling, the concrete is only subjected to the salt crystallization effect of sodium chloride. After 77 dry–wet cycles, the E*_rd_* remains at about 95%, and the mechanical properties of the concrete are well maintained without any signs of expansion or cracking. This indicates that the salt crystallization corrosion effect of NaCl on concrete under dry–wet cycling is very limited.

In the 77 dry–wet cycles of the three solutions, the E*_rd_* of the G60 specimens is generally greater than that of the PC specimens. However, as the number of dry–wet cycles increases, the gap between the two gradually narrows. This indicates that adding slag provides good resistance to long-term salt crystallization corrosion in concrete, but the effect diminishes over time. When compared with the data from solution corrosion, it is found that after 77 dry–wet cycles, the E*_rd_* of both PC and G60 is lower than that under solution corrosion, which corresponds with the mass change trend shown in Figure 10.

**Figure 12:**
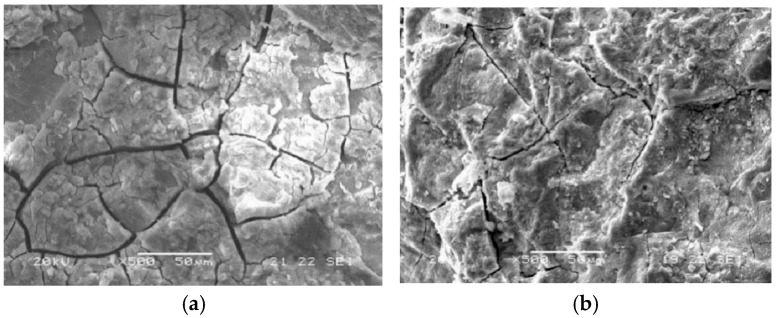
Microcracks in PC and G60 under composite solution salt crystallization corrosion conditions. (**a**) Microcracks of PC under composite solution salt crystallization corrosion; (**b**) Microcracks of G60 under composite solution salt crystallization corrosion.

Furthermore, by scanning the concrete after salt crystallization corrosion using SEM (Figure 12) and comparing it with Figure 7, it can be observed that both PC and G60 specimens under composite solution salt crystallization corrosion have more microcracks with larger widths. Additionally, the surfaces of the specimens under solution corrosion show significantly more severe spalling, which visually demonstrates that under dry–wet cycling, concrete is more affected by the damage caused by the composite solution.

**Figure 13:**
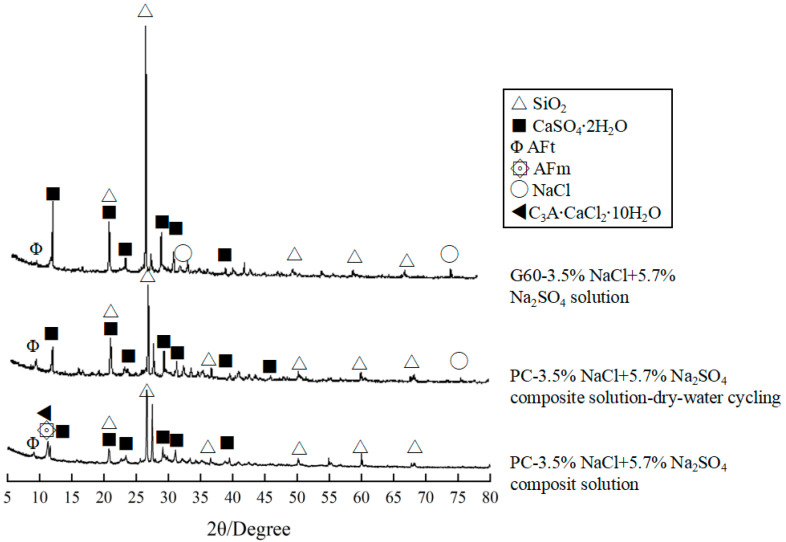
XRD analysis of PC and G60 under chemical corrosion and salt crystallization corrosion conditions in composite solutions.

To further clarify the mechanism of sulfate corrosion, XRD analysis of PC and G60 under chemical corrosion and salt crystallization corrosion conditions in composite solutions has been conducted, as shown in Figure 13. The study reveals that gypsum, ettringite, and AFm, which are expansive substances, are commonly found in concrete after immersion or dry–wet cycling in composite solutions. Additionally, the quantity and types of these substances vary with different corrosive environments.

Taking PC concrete as an example, the quantity of gypsum in the salt crystallization corrosion environment is significantly higher than in the chemical corrosion environment, and the presence of NaCl is detected. This indicates that salt crystallization corrosion causes more severe damage to the concrete, as sulfate and chloride ions in the corrosive medium can easily penetrate the concrete, causing corrosion. In contrast, in chemical corrosion, trace amounts of C_3_A·CaCl_2_·10H_2_O are present in the corrosion products, mainly generated by chloride salt corrosion of the concrete.

In comparison, the amount of gypsum in G60 is higher than in PC, but almost no AFm is formed. Since the formation of AFm occurs after gypsum is exhausted (after being converted from Aft) and consumes C_3_A in the concrete, which negatively impacts the strength of the concrete, its absence in G60 suggests that adding slag significantly improves G60’s resistance to sulfate corrosion.

## 4. Conclusions

In this study, both PC and G60 concrete were subjected to solution corrosion and dry–wet cycling treatments in 3.5% NaCl solution and composite solution (3.5% NaCl + 5.7% Na_2_SO_4_). The corrosion characteristics, mass changes, and relative dynamic elastic modulus variations were monitored over an extended period. The main conclusions are as follows:

(1) Concrete specimens immersed in distilled water and 3.5% NaCl solution for 540 days showed almost no significant damage, with no obvious mass loss or reduction in the relative dynamic elastic modulus. However, in the composite solution, both PC and G60 exhibited significant spalling, with mass losses of 1.266% and 1.403%, and the relative dynamic elastic modulus decreased to 86.992% and 91.380%.
(2) Chloride salt crystallization corrosion only caused mild surface spalling damage. After dry–wet cycling, the specimens’ surfaces showed whitening and powdering phenomena, but both mass and relative dynamic elastic modulus were well maintained. Under composite solution salt crystallization corrosion conditions, both types of concrete exhibited severe surface spalling, resulting in more mass loss and a greater reduction in relative dynamic elastic modulus.
(3) Compared to solution corrosion, dry–wet cycling conditions accelerated the erosion of concrete by the composite solution, leading to greater mass loss and reduction in dynamic elastic modulus. The incorporation of slag improves concrete’s ability to resist ion corrosion in solution corrosion and salt crystallization corrosion, but the effect gradually diminishes with extended soaking time or increased cycling number.

## Figures and Tables

**Table 1:** Chemical composition of cement (mass percentage/%).

Cement	SiO_2_	Al_2_O_3_	CaO	MgO	SO_3_	Fe_2_O_3_	Ignition Loss
P. II 52.5R	20.60	5.03	65.06	0.55	2.24	4.38	1.30

**Table 2:** Physical and mechanical properties of cement.

Specific Surface Area/m^2^·kg^−1^	Setting Time/Min		Flexural Strength/MPa		Compressive Strength/MPa	
	First	Final	3 d	28 d	3 d	28 d
356	108	158	6.0	9.2	27.8	55.7

**Table 3:** Chemical composition of slag (mass percentage/%).

Materials	SiO_2_	Al_2_O_3_	CaO	MgO	SO_3_	Fe_2_O_3_	TiO_2_	K_2_O
SG	32.86	13.21	40.34	2.72	5.59	1.90	1.22	0.94

**Table 4:** Basic physical properties of aggregates.

Aggregate	Bulk Density/ (kg·m^−3^)	Apparent Density/ (kg·m^−3^)	Crushing Value/(%)	Water Absorption Ratio/(%)
Fine sand	1568	2633	-	0.8
Manufactured sand	1640	2953	-	0.9
3~8 mm crushed stones	1634	2932	15.3	0.6
8~16 mm crushed stones	1623	2938	14.5	0.4

**Table 5:** Mix proportions of two different concrete.

Type	Cement/ kg·m^−3^	Slag/kg·m^−3^	Fine Sand/kg·m^−3^	3~8 mm Crushed Stones/kg·m^−3^	8~16 mm Crushed Stones/kg·m^−3^	Water/kg	Superplasticizer/ kg·m^−3^	28 d Compressive Strength/MPa
PC	400	-	736	413	826	152	3.6	58.7
G60	160	240	736	413	826	152	3.2	59.8

Note: G60 refers to a 60% mass proportion of slag in the cementitious materials.

**Table 6:** Mass variation rates under different immersion conditions.

Immersion Time (Days)		0 d/%	30 d/%	60 d/%	120 d/%	180 d/%	270 d/%	330 d/%	360 d/%	390 d/%	460 d/%	500 d/%	540 d/%
Distilled water	PC	0	−0.006	0.002	0.009	−0.018	0.002	0.019	0.032	0.053	0.057	0.028	0.016
	G60	0	−0.016	0.001	0.018	0.012	0.007	0.042	0.066	0.078	0.069	0.053	0.046
3.5% NaCl solution	PC	0	−0.003	0.000	0.002	−0.014	0.018	0.093	0.108	0.122	0.093	0.119	0.083
	G60	0	0.083	0.084	0.085	0.108	0.127	0.173	0.224	0.235	0.204	0.205	0.131
Composite solution	PC	0	0.091	0.114	0.135	0.078	−0.068	−0.404	−0.540	−0.676	−1.021	−1.098	−1.403
	G60	0	−0.050	−0.020	0.010	−0.028	−0.258	−0.373	−0.509	−0.645	−0.865	−0.809	−1.266

**Table 7:** E*_rd_* variation under different immersion solution conditions.

Type/Duration of Corrosive Environment		0 d/%	30 d/%	60 d/%	120 d/%	180 d/%	270 d/%	330 d/%	360 d/%	390 d/%	460 d/%	500 d/%	540 d/%
Distilled water	PC	100	111.911	111.448	110.984	93.137	96.167	94.865	95.799	96.733	106.438	92.763	92.651
	G60	100	116.655	112.394	108.133	95.254	91.042	93.376	101.013	104.650	102.460	106.118	93.788
3.5% NaCl solution	PC	100	109.158	103.112	97.646	100.389	95.462	95.291	95.098	95.155	103.332	112.537	94.91
	G60	100	118.120	115.125	113.343	111.969	99.82	97.163	98.153	117.5757	101.911	119.957	97.847
Composite solution	PC	100	115.573	108.065	97.336	109.956	100.008	89.482	92.563	97.552	86.348	90.501	86.992
	G60	100	114.337	103.056	102.131	107.132	90.918	91.033	94.442	93.392	96.264	91.676	91.380

**Table 8:** Mass change rate under different dry–wet cycling conditions.

Corrosion Environment Type/Dry–Wet Cycle Times		0 Time/%	5 Times/%	8 Times/%	15 Times/%	25 Times/%	38 Times/%	47 Times/%	51 Times/%	56 Times/%	66 Times/%	72 Times/%	77 Times/%
Distilled water	PC	0	−0.041	−0.050	−0.089	−0.128	−0.108	−0.037	−0.050	−0.043	−0.024	−0.070	−0.098
	G60	0	0.032	0.036	0.041	−0.042	0.010	0.037	0.057	0.077	0.061	0.049	0.045
3.5% NaCl solution	PC	0	−0.018	−0.016	−0.003	−0.037	−0.031	0.011	0.045	0.059	0.041	0.051	0.011
	G60	0	0.052	0.056	0.060	0.049	0.081	0.127	0.143	0.160	0.138	0.148	0.129
Composite solution	PC	0.000	0.037	0.056	0.066	0.033	−0.276	−0.681	−0.763	−1.006	−1.285	−1.333	−1.565
	G60	0.000	0.013	0.028	0.013	−0.026	−0.155	−0.321	−0.534	−0.807	−1.151	−1.282	−1.480

**Table 9:** E*_rd_* variation under different dry–wet cycling conditions.

Corrosion Environment Type/Dry–Wet Cycle Times		0 Time/%	5 Times/%	8 Times/%	15 Times/%	25 Times/%	38 Times/%	47 Times/%	51 Times/%	56 Times/%	66 Times/%	72 Times/%	77 Times/%
Distilled water	PC	100.000	100.004	98.954	97.903	93.631	95.875	92.166	87.982	88.797	89.334	90.266	88.081
	G60	100.000	98.741	104.314	109.887	97.995	101.442	95.923	94.908	94.893	95.465	101.205	97.505
3.5% NaCl solution	PC	100.000	108.480	101.192	93.903	91.900	90.049	92.244	90.357	94.469	108.679	103.029	95.245
	G60	100.000	115.814	103.608	91.402	97.878	93.336	93.966	98.135	105.304	110.688	106.891	95.263
Composite solution	PC	100.000	94.277	95.259	96.240	80.388	77.601	80.923	81.098	81.272	82.643	87.317	84.454
	G60	100.000	107.846	100.811	93.776	100.707	84.399	88.12	84.853	81.584	85.192	87.711	84.463

## Data Availability

The data are not publicly available due to privacy restrictions, but the raw data supporting the conclusions of this article will be made available by the authors on request.
